# The Relationship between Social Support, Self-Efficacy and Characteristics of Women with Diabetes during Pregnancy

**DOI:** 10.3390/ijerph19010304

**Published:** 2021-12-28

**Authors:** Grażyna Iwanowicz-Palus, Marta Zarajczyk, Agnieszka Bień, Magdalena Korżyńska-Piętas, Justyna Krysa, Mansur Rahnama-Hezavah, Artur Wdowiak

**Affiliations:** 1 Chair of Obstetrics Development, Faculty of Health Sciences, Medical University of Lublin, 4-6 Staszica St., 20-081 Lublin, Poland; spupalus@gmail.com (G.I.-P.); agnesmbien@gmail.com (A.B.); korzynska.magdalena@gmail.com (M.K.-P.); krysajustyna@gmail.com (J.K.); 2 Chair and Department of Dental Surgery, Medical University of Lublin, 20-081 Lublin, Poland; mansur.rahnama@umlub.pl; 3 Chair of Obstetrics and Gynecology, Faculty of Health Sciences, Medical University of Lublin, 4-6 Staszica St., 20-081 Lublin, Poland; wdowiakartur@gmail.com

**Keywords:** diabetes mellitus, pregnancy, social support, generalized self-efficacy

## Abstract

Background: One of the most common metabolic complications of pregnancy are carbohydrate metabolism disorders resulting in hyperglycemia. The aim of the study was the assessment of impact of socio-demographic variables on the levels of social support and self-efficacy and an investigation of whether there is and how the relationship between social support and self-efficacy is shaped in pregnant women with gestational diabetes. In this study 339 pregnant women with diabetes during pregnancy and 337 healthy pregnant women took part. Methods: The Berlin Social Support Scales (BSSS), the Generalized Self-Efficacy Scale (GSES) and a standardized interview questionnaire were used. Results: The respondents rated Perceived Instrumental Support higher (M = 3.52) than Perceived Emotional Support (M = 3.39). In contrast, Actually Received Support (M = 3.53) was rated higher compared to Support Seeking (M = 2.99) and Need for Support (M = 2.95). The mean generalized self-efficacy score was M = 31.58 in women with diabetes during pregnancy and M = 31.85 in healthy pregnant women. Conclusions: The research results obtained prove the existence of a relationship between GSES and BSSS scores. In pregnant women with diabetes and healthy pregnant women, GSES increases with an increase in perceived support. Additionally, among pregnant women with diabetes, the level of GSES increases with an increase in actually received support. However, in the case of healthy pregnant women, a lower level of need for support is associated with higher level of generalized self-efficacy.

## 1. Introduction

Diabetes is one of the most common metabolic complications which occur in pregnancy. Diabetes in pregnancy can occur as pre-pregnancy diabetes and as hyperglycemia which is diagnosed for the first time in pregnancy. Hyperglycemia first detected during pregnancy, depending on the glycemic levels obtained during a 75 g oral glucose tolerance test (OGTT), is classified as diabetes in pregnancy (DIP) or gestational diabetes mellitus (GDM). It is associated with short-term and long-term complications for both mother and child. Based on 51 studies conducted in 2019 around the world, it has been proven that 20.4 million pregnancies were complicated by various types of hyperglycemia, which is 15.8% of all pregnancies in a given year in the world. It is estimated that 18.3 million pregnant women will be affected by diabetes in 2030–2045. Despite extensive prophylaxis and early detection of carbohydrate metabolism disorders in pregnancy, the problem will be present in the coming decades [1,2].

When a pregnancy is complicated, it becomes difficult for the future mother. Mental health, which is strongly related to family relationships and available social support, is a very important element in the care of people with a chronic disease such as diabetes. The patient’s mental state determines, inter alia, the acceptance level of discomfort associated with the need to cope with the emerging difficulties of everyday life. As a practical matter, the assessment of the availability and perception of social support by individuals makes care for patients, especially in the presence of a chronic disease, more effective [3,4,5,6,7,8,9,10].

There is also evidence of the direct and concomitant effects of social support on individual health, including cardiovascular and immunological health. It has been shown that people with high levels of social support have a lower risk of death compared to those with low levels of support or poorer quality social relationships [11,12].

A complicated pregnancy can be perceived as a difficult situation. The woman may experience anxiety, sadness, and guilt. The method of coping with this situation depends on many factors, including personal psychosocial resilience resources. These are individualized human characteristics which help us cope with difficulties and reduce the impact of experienced stressors. A generalized sense of efficacy is one of such resource [13,14].

Self-efficacy differentiates people in terms of reflecting life’s challenges and intentions about how to act. The stronger an individual’s self-efficacy beliefs, the higher the goals he or she sets, the more persistently he or she pursues them even in the face of difficulties, and strongly engages in achievement despite failures. On the other hand, low self-efficacy is associated with depression, anxiety, helplessness, and unwillingness to demonstrate intentions to act. People who rate their self-efficacy as high, are confident and more often undertake tasks. They are persistent in their actions. However, those who do not believe in their strengths, even if they undertake a challenge, are quickly become discouraged when difficulties occur [15].

Generalized self-efficacy is, therefore, a mental resource which conditions the belief in the ability to take actions aimed at improving or strengthening the health of a pregnant woman through appropriate behaviour. People with a high level of generalized self-efficacy scale are predisposed to setting higher goals in life and pursuing them with greater commitment, also in terms of self-observation and self-care skills in the case of chronic diseases [16,17,18].

### Purpose of the Study

The aim of the study was the assessment of the impact of socio-demographic variables on the levels of social support and self-efficacy and investigation if there is, and how, a relationship between social support and self-efficacy is shaped in pregnant women with gestational diabetes.

## 2. Materials and Methods

### 2.1. Subjects

**Figure 1:**
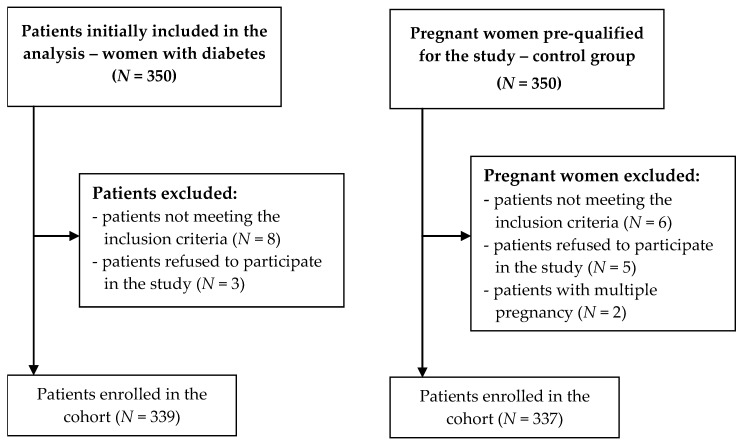
Flowchart of the recruitment process of the patients.

The study was performed in 2016–2017 in Lublin Province, Poland, in a group of 676 pregnant women, of whom 339 had diabetes during pregnancy and were qualified to the study group, and the control group consisted of 337 pregnant women with normal pregnancies – Figure 1. The inclusion criteria were as follows: age above 18 years; consent to participate in the study; single pregnancy, time from diabetes diagnosis exceeding 5 weeks; using health care in Poland throughout the pregnancy; and diabetes diagnosed before or during the current pregnancy as per the current Polish Diabetes Association guidelines:

- pregestational diabetes mellitus (PGDM)—when a woman with diabetes (regardless of type) becomes pregnant;
- gestational diabetes mellitus (GDM) is diagnosed in pregnancy when at least one of the following criteria is met in a 75 g Oral Glucose Tolerance Test (OGTT): fasting glucose 92–125 mg/dL (5.1–6.9 mmol/L), glucose level at 60 min ≥ 180 mg/dL (10 mmol/L) or glucose level at 2 h 153–199 mg/dL (8.5–11 mmol/L);
- diabetes in pregnancy (DIP) is diagnosed in pregnancy when at least one of the following criteria is met: fasting glucose over 126 mg/dL (7 mmol/L), glucose level at 2 h in 75 g OGTT ≥ 200 mg/dL (11.1 mmol/L), or casual glucose level exceeding 200 mg/dL (11.1 mmol/L) with clinical hyperglycemic symptoms [13].

The inclusion criteria for pregnant women in the control group were: age 18 years or older; willingness to participate in the study; single pregnancy; no diabetes in the current pregnancy; and use of health care services in Poland throughout the pregnancy.

The exclusion criteria for both groups were the diagnosis of other diseases which may complicate the pregnancy, such as hypertension, threatened preterm labor, thyroid disease, liver disease, etc., which could affect the perception of quality of life and social support.

The diagnosis of diabetes mellitus was confirmed based on the patient’s medical records. Each patient completed the questionnaire in person, after completing a model form of informed consent for participation in the study.

### 2.2. Data Collection

The study was performed using the diagnostic survey method with questionnaires. The following instruments were used: the Berlin Social Support Scales (BSSS), the Generalized Self-Efficacy Scale (GSES) and a standardized interview questionnaire designed to record the participants’ characteristics.

The BSSS by R. Schwarzer and U. Schutz, adapted into Polish by A. Łuszczyńska and M. Kowalska, comprises six subscales. In the present study, the following subscales were used: I—perceived available support, II—need for support, III—support seeking, V—actually received support, VI—protective buffering (subscale IV—actually provided support was not used).

The names of the scales which were used in the study correspond to the terms of the variables: Perceived Available Support (Emotional and Instrumental) scales refer to the assessment of the availability of help from others, which has a direct impact on health and well-being, regardless of situational factors. The Need for Support scale measures the need to obtain and utilize support from others when a stressful situation arises. The Support Seeking scale is a measure of the frequency of seeking help from others and the Actually Received Support scale measures the perceived help from others reducing the sense of danger in stressful situations. Items are scored on a scale of 1 to 4, where 1 indicates that the respondent considers the statement completely false, and 4 indicates they consider the statement completely true. Higher scores indicate more social support. Cronbach’s α for the questionnaire is 0.80 [19,20].

The GSES developed by R. Schwarzer and M. Jerusalem was adapted into Polish by Z. Juczyński. The scale measures an individual’s general perception of their efficacy in dealing with obstacles and difficult situations on a daily basis. The questionnaire may be used in healthy or ill adult patients, and comprises 10 items. For each item, one of four responses must be selected: 1—not true at all, 2—hardly true, 3—moderately true, 4—exactly true, giving the opportunity to obtain between 1 and 4 points for each of the 10 questions. The total score, ranging between 10 and 40 points, is an indicator of generalized self-efficacy, with higher scores denoting a stronger sense of self-efficacy. Values between 10–24 points are described as low, between 25–29 points as medium, and 30–40 points as a high level of self-efficacy. Cronbach’s α for the questionnaire is 0.85 [21].

### 2.3. Statistical Analyses

Statistical analysis of the collected data was performed using the IBM SPSS Statistics (v. 21) software. For qualitative variables, numbers and percentages in each category were given. Quantitative variables were described using means (M), standard deviations (SD), median (Me) and lower and upper quartile values (Q1 and Q3).

Appropriate statistical procedures were used to verify the hypotheses. The normality of the data distributions was checked using the Shapiro–Wilk test. The Student’s *t*-test (t) for independent groups was used to verify the hypothesis of equality of the means of the studied variable in two populations and one-way ANOVA (F) for independent groups, used to verify the hypothesis of the equality of the means of the studied variable in several populations. In the case of relatively large disproportions between the compared groups and due to the level of measurement of variables (ordinal), non-parametric methods were used. Comparisons between two groups were undertaken using the Mann–Whitney U-test (Z), also called the Wilcoxon Mann–Whitney test. It is used to verify the hypothesis of no significant difference between the median values of the studied variable in two populations (assuming similar variable distributions). Correlations between quantitative variables were analyzed using Pearson’s linear correlation coefficient (r).

A series of regression analyses were also performed, with all explanatory variables introduced in the model. The results were interpreted by comparison of the Beta coefficient (β), in accordance with the correlation strength and direction for each predictor. The variables that were explained included: Perceived Emotional and Instrumental Support (BSSS). The explanatory variables were: age, education, marital status, residence, self-reported living condition, professional activity, number of pregnancies, body mass before pregnancy and weight gain in pregnancy. A dummy coding method was used. This is a method of coding nominal (qualitative) data into numerical data taking values of 0 and 1 for the purpose of statistical analysis, e.g.,: higher education—1 and 0—other education; 1—first pregnancy and 0—second and subsequent pregnancies; 1—BMI in the norm and 0—BMI outside the norm.

The study used a significance threshold of *p <* 0.05. The results are shown to the nearest thousandth (e.g., “0.038”. For values less than 0.001, the notation “<0.001” was used).

### 2.4. Ethics

The study was approved by the Medical University of Lublin Bioethics Committee (decision no. KE-0254/160/2016). Permission was also obtained from each health care institution where the study was performed. Respondents were informed that participation was anonymous, and freely provided their consent to participate. Before the start of the study, they were also informed that any findings would only be used for research purposes.

## 3. Results

Among pregnant women with diabetes during pregnancy, the majority were respondents aged 26–30 (31.9%), married (88.5%), living in a urban—province capital city (39.5%), having a master’s degree (43.1%), professionally active (61.1%), assessing their living conditions as good (53.1%), being in their first pregnancy (37.8%), with normal body weight before pregnancy (51.1%) and the weight gain during pregnancy oscillated between 7–10 kg (38.1%).

In the control group, the majority were pregnant women in the age range of 26–30 (35.0%), in a relationship (89.6%), inhabitants of rural areas (36.5%), with a master’s degree (44.2%), professionally active (56.7%), assessing their living conditions as good (52.8%), being in their second pregnancy (37.7%), with normal body weight before pregnancy (68.0%) and the weight gain during pregnancy oscillated between 11–16 kg (33.8)—Table 1.

There were statistically significant differences (*p* < 0.05) between the control group and the study group in terms of the need for support and the currently received support. Pregnant women with diabetes during pregnancy showed less need for support and currently received support compared to the group of women who did not have diabetes during pregnancy—Table 2.

The analysis did not show any significant statistical dependence (*p* > 0.05) between social support and the age of the respondents with diabetes during pregnancy and healthy pregnant women.

Both in the relation to study group and control group, a statistically significant relationship was demonstrated between the education of pregnant women and the perceived available emotional and instrumental support as well as the currently received support. In contrast, in the study group differences were also shown in the search for support (*p* < 0.05). In both groups the highest results in the above scales were found among pregnant women with higher education.

The results show a statistically significant correlation (*p* < 0.05) between the perceived available emotional and instrumental support in women with diabetes during pregnancy and marital status. Higher results were observed in the group of women who were in a relationship. The results of the study did not show any statistically significant correlation in this field in the group of healthy pregnant women.

The results of the study showed statistically significant differences (*p* < 0.05) between the perceived available emotional and instrumental support and the place of residence. It was the inhabitants of the voivodeship city that obtained the highest results in these scales, and the lowest were obtained by the inhabitants of cities other than voivodeships. On the other hand, in the case of the need for support, seeking support and currently received support, the highest results were recorded among women living in rural areas, and the lowest results were also found among women living in cities other than voivodeships. However, in the case of healthy pregnant women, no statistically significant correlation was found between social support and the place of residence.

A statistically significant relationship (*p* < 0.05) was observed in terms of the assessment of living conditions. In the case of the need for support, the highest results were obtained by pregnant women with diabetes, who assessed the living conditions as good, while in the case of other scales, the highest results were reported by pregnant women with diabetes, who assessed the living conditions as very good. In the case of the control group, significant statistical relationships were demonstrated in terms of perceived available emotional and instrumental support and currently received support. It was healthy pregnant women who assessed their living conditions as very good and showed the highest level of social support.

Professionally active pregnant women with diabetes, compared to non-working women (*p* < 0.05), showed higher results in terms of perceived available emotional and instrumental support as well as seeking and currently receiving support. Among healthy pregnant women, similar relationships were found only in terms of perceived available emotional and instrumental support.

Analyzing the relationship between social support and the number of pregnancies the authors found that the level of perceived available emotional and instrumental support and the seeking support decreased with the next pregnancy in pregnant women with diabetes during pregnancy, and this relation was not shown in the case of healthy pregnant women.

Statistically significant relationships (*p* < 0.05) among women with diabetes during pregnancy were also shown between the level of social support and body weight before pregnancy. The lowest level of perceived available instrumental support, the need for support, and seeking support were found in obese women compared to overweight, normal or underweight women from before the pregnancy. In the case of healthy pregnant women, correlations in terms of perceived available emotional support and currently received support were noticed—similarly to the group of pregnant women with diabetes during pregnancy, and the lowest values were reported by women who were obese before pregnancy.

In the case of weight gain during pregnancy, the diabetic pregnant women who declared the lowest gain of extra kilograms during pregnancy showed the highest level in terms of perceived available emotional and instrumental support and currently received support. Healthy pregnant women, who put on weight over 16 kg during pregnancy showed the highest level of need for support. The results of the study showed that the highest level of perceived social support in pregnant women was observed among patients with higher education, being in a relationship, living in provincial cities, declaring better living conditions, working professionally, with normal pre-pregnancy body weight, or being underweight and having the smallest weight gain during pregnancy—Table 3.

Comparing the generalized self-efficacy between the study group and the control group, no statistically significant difference (*p* > 0.05) was found—Table 4.

The statistical analysis showed a statistically significant correlation between education and general self-efficacy in women with diabetes during pregnancy. Pregnant women with a master’s degree were characterized by the highest self-efficacy level, while the lowest level was represented by the respondents with secondary education (*p* = 0.046). A statistically significant relationship was also found between the assessment of living conditions and the general sense of self-efficacy among healthy pregnant women and women with diabetes during pregnancy. The higher level of generalized self-efficacy in both groups was associated with a better assessment of living conditions (*p* < 0.001). A statistically significant relationship was also noticed in the scope of the declared body weight before pregnancy in the group of healthy pregnant women. The highest values of generalized self-efficacy were declared by overweight women before pregnancy. The results of the study show that pregnant women who report very good living conditions, pregnant women with diabetes during pregnancy who have a master’s degree, and healthy overweight before pregnancy women showed the highest level of general self-efficacy, i.e., they belonged to the group of people who can best deal with difficult situations in life—Table 5.

The regression model for the Perceived Emotional Support and Perceived Instrumental Support is shown in Table 6. Statistically significant predictors for the Perceived Emotional Support included: higher education (*β* = 0.141 *p* = 0.010), being married (*β* = 0.206; *p* = 0.000), having very good/good living conditions (*β* = 0.125; *p* = 0.019), being primiparous (*β* = 0.178; *p* = 0.001), having a normal weight before pregnancy (*β* = 0.138; *p* = 0.007), and having a pregnancy weight gain of less than 10 kg (*β* = −0.224; *p* = 0.000). Multilevel variable scanning showed that higher Perceived Instrumental Support characterized women who had a college degree *(β* = 0.152; *p* = 0.006), were married (*β* = 0.193; *p* = 0.000), rated their living conditions as good/very good (*β* = 0.162; *p* = 0.003), were primiparous (*β* = 0.121; *p* = 0.027), had a normal pre-pregnancy weight (*β* = 0.117; *p* = 0.021), and pregnancy weight gain was less than 10 kg (*β* = −0.204; *p* = 0.000).

The correlation between generalized self-efficacy and social support in women with diabetes during pregnancy and healthy pregnant women showed a positive relationship of average strength. With the increase in self-efficacy, the level of perceived available emotional and instrumental support and currently received support (*p* < 0.001) among pregnant women with diabetes increases. In the group of healthy pregnant women, a negative correlation was also shown in terms of the need for support. Along with the increase in self-efficacy, the need for social support in healthy pregnant women decreases (*p* = 0.001)—Table 7.

## 4. Discussion

The studies conducted to date show a positive impact of social support on various aspects of physical and mental health, as well as coping with difficult situations, thus improving the general well-being of people in new and often stressful moments of life [9,10,22]. Despite these relationships, research on women with diabetes during pregnancy still comprise a small number of studies on social support [9]. Comparing in our own research the levels of social support in the group of women with diabetes during pregnancy and healthy pregnant women, the higher currently received support was declared by healthy pregnant women and, moreover, they also showed a higher need for a social support.

In the results of our study, pregnant women with diabetes and healthy pregnant women with higher education had the highest scores in the scale of perceived available emotional, instrumental and currently received social support, in the case of women with diabetes also in terms of seeking support. The results obtained are consistent with studies conducted among pregnant women by Azimi et al. (2018) and Abdollahpour et al. (2015) [23,24]. No correlation between the education of primiparous women and social support was demonstrated by Nazari et al. (2015) [25]. However, in the studies by Ahmed et al. (2017), a higher level of social support correlated with a lower level of education [10].

Marital status was one of the socio-demographic factors influencing the level of social support among pregnant women with diabetes. The authors’ own research showed a relationship between the marital status of women with diabetes during pregnancy and the perceived available emotional and instrumental support. Women who were in a relationship had higher scores in both scales of the BSSS questionnaire. Numerous reports on the positive impact of support received from family members, including mainly the husband, on the well-being and health of pregnant women, pregnant women with diabetes and people with type 1 and 2 diabetes can be found in the literature [22,24,25,26,27]. Married women can rely on their husbands who can help them with self-care or self-care during their illness.

Women with diabetes during pregnancy, residents of cities other than voivodeships, declared the lowest values of social support in all scales of the BSSS questionnaire, while the highest perceived available emotional and instrumental support was reported by residents of voivodeship cities. Higher perceived available support in these scales may be associated with a better infrastructure of voivodeship cities, which gives better access to e.g., medical advice and specialists. On the other hand, the highest values in terms of the demand for support, as well as the search for and currently received support, were found in rural residents. The results of our own research correspond with the research of Edmonds et al. (2011) and Ahmed et al. (2017), who claimed that better interpersonal contacts, giving a sense of greater social support, are the nature of people living in rural areas [10,27]. People living there offer their help more often, do not feel embarrassed to seek it, and appreciate help received from someone more [10].

In our study, higher social support in all scales of social support was declared by women with diabetes who assessed their living conditions better. Mirabzadeh et al. (2013) [28] and Azimi et al. (2018) share a similar opinion, showing smaller social support networks among primiparous women with lower incomes [23]. In turn, in the studies by Ahmed et al. (2017), pregnant women with average income showed a higher level of social support [10]. Studies conducted by Abdollahpour et al. (2015), Nazari et al. (2015) and Shishehgar et al. (2015), showed no correlation between living conditions and social support among pregnant women [24,25,26]. According to Nazari et al. (2015), the lack of relationship between social support and living conditions may result from the fact that perceived social support is a very subjective feeling, depending on the level of perception, and not the generally accepted measure, which is the assessment of the financial situation [25]. These research results partially correspond with the results of the authors’ research, in which, among healthy pregnant women, no statistically significant dependence was noticed in terms of the need for support and seeking social support.

Working women who belonged to the study group of pregnant women with diabetes during pregnancy rated higher perceived available emotional and instrumental support, as well as seeking support and currently receiving support. It is probable that working women are more busy and burdened with more responsibilities, hence they need more support from others than non-working mothers.

The highest values in the field of perceived available emotional and instrumental support in our own research were shown by women with diabetes during their first pregnancy. The results of own research are identical to the results presented by Gebuza et al. (2016) assessing social support among pregnant women [7]. Study results may be dictated by the fact that inexperienced mothers, primiparous women, feel more loved and cared for by their husbands, and they can count on their help to a greater extent than mothers of two or more children.

Hyperglycemic pregnant women who declared the lowest value of extra kilos during pregnancy showed the highest level in terms of perceived available emotional and instrumental support and currently received support. However, among healthy pregnant women, women with weight gain over 16 kg during pregnancy showed the highest level of need for support. Similar conclusions presented Marquez et al. (2016) and Mirabzadeh et al. (2013), who proved that people declaring a higher level of social support are characterized by easier weight loss, which translates into a better perception of their own health [4,28].

A generalized sense of self-efficacy is an element which determines an individual’s health. People who present a high level of generalized self-efficacy scale predispose to setting higher goals in life and pursuing them with greater commitment, also in terms of self-observation and self-care skills in the case of chronic diseases [16,17,18].

In the authors’ study, the average assessment of the generalized sense of self-efficacy was lower among pregnant women with diabetes compared to healthy pregnant women. The GSES values in both groups were within the high reference values of the scale, as in the studies by Rydlewska A et al. (2013) among patients with cardiac insufficiency and in the studies by Rogal D. et al. (2015) among women after hysterectomy [29,30]. On the other hand, in the studies conducted by Bień et al. (2021) among obese women with a risk of preterm labor, it was shown that the mean GSES score was 28.02 and was within the upper limits of the mean reference values, as in the study by Rzońca et al. (2018) among women with polycystic ovary syndrome [31,32]. The high self-efficacy score translates into a better ability to assess the life situation, more effective coping with difficulties and obstacles, as well as greater involvement in the therapeutic process, adjusting to medical recommendations and the need to change one’s lifestyle [33].

In the studies by D’Souz et al. (2017), elderly people showed a higher level of generalized self-efficacy compared to younger people [17]. In the study of Brunton et al. (2020), the self-efficacy of mothers correlated with the level of pregnancy acceptance, but, similarly to the authors’ own research, it was not related to age [34]. It is possible that the lack of this relationship among pregnant women was caused by a lower age difference between the respondents, compared to the previously analyzed results of studies in which this relationship was statistically significant.

The results of the study showed a correlation between the education of the pregnant woman and a general sense of self-efficacy. Women with higher education showed a higher level of motivation. The results of the study corresponded with the results of the research by D’Souz et al. (2017), where patients with type 1 diabetes who declared better education had a higher level of generalized self-efficacy [17].

In the Kav et al. (2017) [18] study people with type 2 diabetes who were not in a relationship and did not work had a high sense of self-efficacy. Perhaps single people, who cannot expect help of a loved one in many life situations, had to adapt to life by mobilizing their own resources to take appropriate action.

In our study the assessment of living conditions turned out to be another sociodemographic factor which influences the level of generalized self-efficacy. Women who assessed their living conditions to be better showed a stronger conviction in their own effectiveness. The correlation was also showed by D’Souza et al. (2017) and Imes et al. (2016) who conducted a study among patients with type 1 diabetes [17,35].

Our study also showed that healthy pregnant women with overweight and normal body weight before pregnancy declared higher self-efficacy compared to obese or underweight pregnant women. As in the studies by Rzońca et al. (2018), a lower GSES level was declared by women with a higher BMI [32]. On the other hand, studies by Sekuła et al. (2018) showed no effect of BMI on the level of self-efficacy [36].

The authors also noted a correlation between the generalized sense of self-efficacy and the social support of women with diabetes during pregnancy. A high level of generalized self-efficacy was associated with a higher perceived available emotional and instrumental support, as well as currently received support. In healthy pregnant women, the need for social support decreases as the sense of self-efficacy increases. However, in the study of Peimani et al. (2018), among people with type 2 diabetes, a lower level of generalized self-efficacy was observed in the group of people supported by peers [37].

The study highlights the need to provide holistic care for healthy pregnant women and pregnant women with diabetes, ensuring not only medical care, but also paying special attention to psychosocial aspects. The study results may indicate in which aspects of caring for women with normal pregnancy and complicated by diabetes, special procedures should be implemented in order to ensure comprehensive medical and psychosocial care that ensure better treatment and care. Such activities may include, for example, the provision of care and extended health education by family midwives, the participation of pregnant women in organized support groups and, in the case of women with diabetes during pregnancy, the provision of ongoing endocrine, diabetes care and nutrition therapy.

The study of social support and self-efficacy among pregnant women with diabetes increases the scope of available knowledge about the psychological potential of somatic health in women with diabetes during pregnancy. Appropriate conduct of a therapeutic team who knows the individual patient needs and groups for whom additional social support should be provided can optimize obstetric care for women with diabetes in pregnancy. Taking into account the study results on the level of perceived social support in women with diabetes during pregnancy, special attention should be paid to the strengthening of the sense of social support among pregnant diabetic women who have primary and vocational education, are single, assess their living conditions as poor, multiparas with obesity before pregnancy and those with the greatest weight gain in pregnancy, because these groups of women declare the lowest level of perceived support.

## 5. Conclusions

Higher perceived emotional and instrumental support was observed in pregnant women with diabetes during pregnancy who had a college degree, were married, reported better living conditions, were pregnant for the first time, with normal BMI before pregnancy, and those with the least weight gain in pregnancy. Higher levels of need for support were shown by women living in rural areas, with good self-reported living conditions and with normal BMI before pregnancy. Support seeking of pregnant women with diabetes is determined by their education, place of residence, living conditions, work activity, pregnancy sequence, and body weight before the pregnancy. The level of actually received support of pregnant women with diabetes depends on their education, place of residence, living conditions, work activity, and weight gain during pregnancy. The self-efficacy of pregnant women with diabetes is associated with higher education and better social and living conditions. With the increase of perceived available emotional and instrumental support and currently received support, the level of self-efficacy among pregnant women with diabetes grows.

## Figures and Tables

**Table 1:** Socio-demographic characteristics of the women in the study.

Characteristics		Case Group *N* (%)	Control Group *N* (%)
		339 (50.8)	337 (49.2)
Age	18–25 y/o	60 (17.7)	91 (27.0)
	26–30 y/o	108 (31.9)	118 (35.0)
	31–35 y/o	99 (29.2)	89 (26.4)
	≥36 y/o	72 (21.2)	39 (11.6)
Education	Primary	32 (9.4)	35 (10.4)
	High school	88 (26.0)	110 (32.6)
	Vocational/college degree	73 (21.5)	43 (12.8)
	Master’s degree	146 (43.1)	149 (44.2)
Marital status	Married	300 (88.5)	302 (89.6)
	Single	39 (11.5)	35 (10.4)
Residence	Urban—province capital	134 (39.5)	109 (32.3)
	Urban—other	106 (31.3)	105 (31.2)
	Rural	99 (29.2)	123 (36.5)
Self-reported living conditions	Very good	101 (29.8)	131 (38.9)
	Good	180 (53.1)	178 (52.8)
	Average	58 (17.1)	28 (8.3)
Professional activity	Professionally active	207 (61.1)	191 (56.7)
	Professionally inactive	132 (38.9)	146 (43.3)
Number of pregnancies	First pregnancy	128 (37.8)	117 (34.7)
	Second pregnancy	117 (34.5)	127 (37.7)
	≥3 pregnancy	94 (27.7)	93 (27.6)
Body mass before pregnancy	Underweight	20 (5.9)	32 (9.5)
	Normal weight	173 (51.1)	229 (68.0)
	Overweight	114 (33.6)	64 (18.9)
	Obesity	32 (9.4)	12 (3.6)
Weight gain in pregnancy	Over 16 kg	43 (12.7)	76 (22.6)
	11–16 kg	101 (29.8)	114 (33.8)
	7–10 kg	129 (38.1)	111 (32.9)
	Less than 6 kg	66 (19.5)	36 (10.7)

**Table 2:** The comparative analysis of social support in the study and control group.

Berlin Social Support Scales (BSSS)	Case Group					Control Group					Statistical Analysis	
	M	SD	Q1	Me	Q3	M	SD	Q1	Me	Q3	t	*p*
Perceived Emotional Support	3.39	0.51	3.00	3.50	3.75	3.46	0.52	3.25	3.50	4.00	−1.760	0.079
Perceived Instrumental Support	3.52	0.58	3.00	3.75	4.00	3.60	0.53	3.13	4.00	4.00	−1.856	0.064
Need for Support	2.95	0.53	2.50	3.00	3.25	3.06	0.51	2.75	3.00	3.50	−2.855	0.004
Support Seeking	2.99	0.66	2.60	3.00	3.40	3.00	0.63	2.60	3.00	3.40	−0.191	0.849
Actually Received Support	3.53	0.53	3.20	3.80	4.00	3.63	0.50	3.40	3.80	4.00	−2.482	0.013

M—mean; SD—standard deviation; Me—median; Q1—lower quartile; Q3—upper quartile.

**Table 3:** The analysis of the relationship between social support and sociodemographic variables among pregnant women.

Sociodemographic Variables		Berlin Social Support Scales (BSSS)									
		Perceived Emotional Support		Perceived Instrumental Support		Need for Support		Support Seeking		Actually Received Support	
		Case Group M (SD)	Control Group M (SD)	Case Group M (SD)	Control Group M (SD)	Case Group M (SD)	Control Group M (SD)	Case Group M (SD)	Control Group M (SD)	Case Group M (SD)	Control Group M (SD)
Age	18–25 y/o	3.33 (0.56)	3.46 (0.55)	3.46 (0.63)	3.60 (0.52)	2.98 (0.46)	3.03 (0.52)	3.08 (0.60)	2.91 (0.66)	3.43 (0.64)	3.60 (0.52)
	26–30 y/o	3.36 (0.54)	3.47 (0.51)	3.46 (0.60)	3.61 (0.52)	2.92 (0.49)	3.12 (0.48)	2.97 (0.64)	3.04 (0.67)	3.52 (0.56)	3.68 (0.45)
	31–35 y/o	3.46 (0.46)	3.44 (0.52)	3.66 (0.52)	3.57 (0.56)	3.03 (0.64)	3.01 (0.53)	3.03 (0.64)	2.99 (0.56)	3.64 (0.41)	3.61 (0.56)
	≥36 y/o	3.41 (0.51)	3.49 (0.45)	3.47 (0.59)	3.65 (0.52)	2.86 (0.56)	3.07 (0.50)	2.88 (0.74)	3.10 (0.57)	3.49 (0.54)	3.59 (0.46)
	Statistical analysis	*p* = 0.341	*p* = 0.965	*p* = 0.052	*p* = 0.895	*p* = 0.191	*p* = 0.441	*p* = 0.275	*p* = 0.322	*p* = 0.094	*p* = 0.623
Education	Primary	3.17 (0.54)	3.32 (0.60)	3.44 (0.54)	3.56 (0.57)	2.88 (0.44)	3.05 (0.53)	2.78 (0.79)	2.79 (0.80)	3.35 (0.50)	3.58 (0.60)
	High school	3.34 (0.54)	3.35 (0.61)	3.36 (0.69)	3.51 (0.60)	3.01 (0.46)	3.08 (0.52)	3.02 (0.66)	3.01 (0.67)	3.44 (0.61)	3.56 (0.55)
	Vcational/college degree	3.27 (0.56)	3.46 (0.51)	3.39 (0.62)	3.53 (0.55)	2.87 (0.48)	2.93 (0.43)	2.87 (0.62)	2.87 (0.47)	3.42 (0.60)	3.46 (0.64)
	Master’s degree	3.54 (0.40)	3.58 (0.38)	3.70 (0.44)	3.70 (0.44)	2.97 (0.59)	3.09 (0.51)	3.07 (0.62)	3.07 (0.58)	3.69 (0.41)	3.75 (0.34)
	Statistical analysis	*p* < 0.001	*p* = 0.001	*p* = 0.001	*p* = 0.025	*p* = 0.270	*p* = 0.304	*p* = 0.036	*p* = 0.054	*p* < 0.001	*p* = 0.001
Marital status	Married	3.43 (0.48)	3.46 (0.51)	3.56 (0.57)	3.61 (0.51)	2.95 (0.54)	3.07 (0.51)	2.99 (0.66)	3.01 (0.61)	3.57 (0.49)	3.65 (0.48)
	Single	3.15 (0.61)	3.49 (0.53)	3.24 (0.58)	3.49 (0.64)	2.97 (0.44)	2.99 (0.51)	2.95 (0.64)	2.87 (0.78)	3.26 (0.75)	3.49 (0.63)
	Statistical analysis	*p* = 0.009	*p* = 0.667	*p* = 0.001	*p* = 0.285	*p* = 0.648	*p* = 0.286	*p* = 0.656	*p* = 0.369	*p* = 0.052	*p* = 0.110
Residence	Urban—province capital	3.50 (0.42)	3.48 (0.60)	3.61 (0.51)	3.63 (0.54)	2.96 (0.53)	3.07 (0.47)	3.05 (0.64)	2.98 (0.67)	3.60 (0.45)	3.72 (0.40)
	Urban—other	3.22 (0.55)	3.48 (0.44)	3.37 (0.67)	3.57 (0.52)	2.81 (0.50)	3.01 (0.56)	2.83 (0.68)	2.98 (0.54)	3.35 (0.61)	3.58 (0.52)
	Rural	3.44 (0.51)	3.44 (0.49)	3.56 (0.56)	3.60 (0.53)	3.08 (0.52)	3.10 (0.48)	3.07 (0.63)	3.02 (0.66)	3.64 (0.51)	3.59 (0.55)
	Statistical analysis	*p* < 0.001	*p* = 0.772	*p* = 0.005	*p* = 0.749	*p* = 0.001	*p* = 0.443	*p* = 0.012	*p* = 0.819	*p* < 0.001	*p* = 0.060
Self-reported living conditions	Very good	3.58 (0.55)	3.57 (0.51)	3.77 (0.49)	3.68 (0.51)	2.99 (0.53)	2.99 (0.47)	3.06 (0.62)	3.10 (0.62)	3.72 (0.53)	3.67 (0.52)
	Good	3.40 (0.57)	3.35 (0.57)	3.51 (0.56)	3.52 (0.53)	3.00 (0.55)	3.05 (0.51)	3.04 (0.64)	3.08 (0.65)	3.55 (0.57)	3.59 (0.54)
	Average/poor	3.06 (0.58)	3.02 (0.63)	3.12 (0.61)	3.12 (0.61)	2.75 (0.57)	2.97 (0.54)	2.69 (0.71)	3.05 (0.68)	3.15 (0.61)	3.36 (0.62)
	Statistical analysis	*p* < 0.001	*p* < 0.001	*p* < 0.001	*p* < 0.001	*p* = 0.005	*p* = 0.221	*p* = 0.001	*p* = 0.164	*p* < 0.001	*p* = 0.001
Professional activity	Professionally active	3.45 (0.47)	3.53 (0.45)	3.61 (0.55)	3.66 (0.50)	2.99 (0.55)	3.10 (0.50)	3.08 (0.64)	3.04 (0.60)	3.59 (0.50)	3.64 (0.49)
	Professionally inactive	3.30 (0.55)	3.37 (0.58)	3.39 (0.61)	3.52 (0.56)	2.88 (0.48)	3.02 (0.51)	2.85 (0.65)	2.94 (0.66)	3.44 (0.58)	3.62 (0.51)
	Statistical analysis	*p* = 0.010	0.005	*p* = 0.001	0.021	*p* = 0.059	0.177	*p* = 0.001	0.184	*p* = 0.011	0.729
Number of pregnancies	First pregnancy	3.51 (0.49)	3.50 (0.42)	3.63 (0.54)	3.60 (0.49)	3.01 (0.54)	3.12 (0.52)	3.15 (0.61)	3.04 (0.59)	3.60 (0.53)	3.70 (0.37)
	Second pregnancy	3.34 (0.48)	3.45 (0.54)	3.47 (0.56)	3.57 (0.58)	2.88 (0.48)	3.03 (0.48)	2.94 (0.62)	2.96 (0.62)	3.54 (0.49)	3.60 (0.60)
	≥3 pregnancy	3.30 (0.53)	3.43 (0.58)	3.43 (0.65)	3.64 (0.51)	2.96 (0.55)	3.03 (0.52)	2.83 (0.71)	2.99 (0.69)	3.43 (0.58)	3.58 (0.49)
	Statistical analysis	*p* = 0.003	*p* = 0.577	*p* = 0.022	*p* = 0.619	*p* = 0.135	*p* = 0.272	*p* = 0.001	*p* = 0.597	*p* = 0.077	*p* = 0.149
Body mass before pregnancy	Underweight	3.34 (0.63)	3.26 (0.53)	3.64 (0.48)	3.55 (0.52)	2.91 (0.27)	2.96 (0.42)	3.02 (0.54)	2.94 (0.47)	3.38 (0.65)	3.52 (0.53)
	Normal weight	3.46 (0.49)	3.53 (0.47)	3.59 (0.55)	3.64 (0.52)	3.06 (0.51)	3.10 (0.51)	3.15 (0.62)	3.04 (0.66)	3.58 (0.50)	3.69 (0.43)
	Overweight	3.37 (0.49)	3.39 (0.57)	3.44 (0.63)	3.50 (0.55)	2.88 (0.54)	2.97 (0.55)	2.85 (0.67)	2.85 (0.55)	3.56 (0.51)	3.52 (0.65)
	Obesity	3.19 (0.51)	3.21 (0.74)	3.43 (0.57)	3.50 (0.56)	2.70 (0.57)	3.04 (0.35)	2.64 (0.57)	3.07 (0.56)	3.30 (0.67)	3.50 (0.57)
	Statistical analysis	*p* = 0.339	*p* = 0.002	*p* = 0.040	*p* = 0.130	*p* = 0.007	*p* = 0.212	*p* = 0.001	*p* = 0.216	*p* = 0.201	*p* = 0.007
Weight gain in pregnancy	Over 16 kg	3.21 (0.65)	3.45 (0.46)	3.27 (0.73)	3.57 (0.52)	2.94 (0.54)	3.18 (0.47)	2.86 (0.70)	3.09 (0.57)	3.44 (0.57)	3.64 (0.46)
	11—16 kg	3.29 (0.52)	3.51 (0.47)	3.43 (0.63)	3.66 (0.47)	2.94 (0.55)	3.11 (0.50)	2.98 (0.64)	3.04 (0.59)	3.38 (0.58)	3.73 (0.46)
	7—10 kg	3.48 (0.44)	3,48 (0.52)	3.59 (0.52)	3.58 (0.57)	2.99 (0.49)	2.99 (0.52)	3.05 (0.63)	2.93 (0.67)	3.60 (0.50)	3.58 (0.52)
	≤6 kg	3.51 (0.43)	3.28 (0.69)	3.68 (0.45)	3.51 (0.59)	2.89 (0.56)	2.90 (0.49)	2.96 (0.69)	2.86 (0.69)	3.69 (0.42)	3.48 (0.60)
	Statistical analysis	*p* = 0.002	*p* = 0.726	*p* = 0.006	*p* = 0.373	*p* = 0.682	*p* = 0.030	*p* = 0.234	*p* = 0.187	*p* = 0.007	*p* = 0.007

**Table 4:** The comparative analysis of the generalized self-efficacy between the study group and the control group.

Generalized Self-Efficacy (GSES)	M	SD	Min	Max	Q1	Me	Q3	Statistical Analysis	
								t	*p*
Case group	31.58	4.60	18.00	40.00	30.00	30.00	34.00	−0.807	0.420
Control group	31.85	4.31	18.00	40.00	30.00	31.00	34.00		

M—mean; SD—standard deviation; Me—median; Q1—lower quartile; Q3—upper quartile.

**Table 5:** The analysis of the relationship between the sense of self- efficacy and sociodemographic variables in pregnant women.

Sociodemographic Variables		Case Group M (SD)	Statistical Analysis	Control Group M (SD)	Statistical Analysis
Age	18–25 y/o	31.13 (4.82)	*p* = 0.249	31.75 (3.92)	*p* = 0.937
	26–30 y/o	31.07 (4.70)		31.99 (4.13)	
	31–35 y/o	32.25 (4.62)		31.69 (4.43)	
	≥36 y/o	31.78 (4.16)		32.08 (5.42)	
Education	Primary	31.94 (4.74)	*p* = 0.046	31.23 (4.66)	*p* = 0.763
	High school	30.40 (4.65)		31.74 (4.38)	
	Vocational/college degree	31.84 (4.54)		32.05 (4.34)	
	Master’s degree	32.08 (4.48)		32.03 (4.18)	
Marital status	Married	31.67 (4.45)	*p* = 0.603	31.83 (4.26)	*p* = 0.560
	Single	30.85 (5.59)		32.06 (4.76)	
Residence	Urban—province capital	31.31 (4.36)	*p* = 0.581	32.08 (4.44)	*p* = 0.392
	Urban—other	31.57 (4.92)		32.11 (4.31)	
	Rural	31.95 (4.57)		31.43 (4.18)	
Self-reported living conditions	Very good	31.95 (4.73)	*p* < 0.001	32.05 (4.33)	*p* < 0.001
	Good	31.24 (4.67)		31.17 (4.55)	
	Average	30.36 (4.46)		30.40 (4.56)	
Professional activity	Professionally active	31.65 (4.65)	*p* = 0.711	32.02 (4.37)	*p* = 0.418
	Professionally inactive	31.46 (4.53)		31.64 (4.23)	
Number of pregnancies	First pregnancy	31.48 (4.84)	*p* = 0.660	31.35 (4.11)	*p* = 0.256
	Second pregnancy	31.88 (4.73)		32.25 (4.33)	
	≥3 pregnancy	31.33 (4.08)		31.95 (4.50)	
Body mass before pregnancy	Underweight	31.75 (4.31)	*p* = 0.872	30.34 (3.16)	*p* = 0.044
	Normal weight	31.72 (4.57)		32.10 (4.37)	
	Overweight	31.49 (4.90)		32.17 (4.69)	
	Obesity	31.19 (3.91)		31.00 (2.95)	
Weight gain in pregnancy	Over 16 kg	30.67 (4.45)	*p* = 0.188	31.11 (3.96)	*p* = 0.161
	11–16 kg	31.00 (4.83)		32.32 (4.24)	
	7–11 kg	31.91 (4.58)		31.83 (4.48)	
	Less than 6 kg	32.39 (4.24)		32.06 (4.60)	

M—mean, SD—standard deviation; y/o—years old.

**Table 6:** Regression analysis of perceived social support and sociodemographic variables.

Predyktor	Berlin Social Support Scales (BSSS)									
	Perceived Emotional Support F = 9.510; *p* < 0.001; R = 0.454; R^2^ = 0.206					Perceived Instrumental Support F = 9.407; *p* < 0.001; R = 0.452; R^2^ = 0.205				
	B	SE	*β*	t	*p*	B	SE	*β*	t	*p*
Age	0.005	0.026	0.011	0.195	0.846	−0.030	0.030	−0.058	0.195	0.846
Education ^A^	0.144	0.056	0.141	2.579	0.010	0.179	0.064	0.152	2.770	0.006
Marital status ^B^	0.326	0.086	0.206	3.808	0.000	0.352	0.099	0.193	3.553	0.000
Residence ^C^	−0.034	0.056	−0.031	−0.606	0.545	−0.042	0.065	−0.033	−0.646	0.518
Self-reported living condition ^D^	0.138	0.059	0.125	2.349	0.019	0.206	0.068	0.162	3.040	0.003
Professional activity ^E^	0.018	0.056	0.018	0.326	0.745	0.085	0.065	0.071	1.307	0.192
Number of pregnancies ^F^	0.185	0.056	0.178	3.280	0.001	0.145	0.065	0.121	2.226	0.027
Body mass before pregnancy ^G^	0.139	0.051	0.138	2.728	0.007	0.137	0.059	0.117	2.320	0.021
Weight gain in pregnancy ^H^	−0.229	0.052	−0.224	−4.407	0.000	−0.240	0.060	−0.204	−4.004	0.000

*β*—standardized coefficients; *SE*—bootstrapped standard errors. Reference categories: ^A^ 1—Higher education; ^B^ 1—Married; ^C^ 1—Urban; ^D^ 1—Good/very good; ^E^ 1—Professionally active; ^F^ 1—First pregnancy; ^G^ 1—Normal weight; ^H^ 1—Over 10 kg.

**Table 7:** The correlation between generalized self-efficacy and social support of women with diabetes during pregnancy.

Berlin Social Support Scales (BSSS)	Generalized Self-Efficacy (GSES)			
	Case Group		Control Group	
	r	*p*	r	*p*
Perceived Emotional Support	0.314	<0.001	0.286	<0.001
Perceived Instrumental Support	0.303	<0.001	0.237	<0.001
Need for Support	−0.052	0.337	−0.174	0.001
Support Seeking	0.041	0.455	0.068	0.210
Actually Received Support	0.329	<0.001	0.151	0.006

r—Pearson’s linear correlation coefficient.

## Data Availability

The datasets generated during and/or analysed during the current study are available from the corresponding author on reasonable request.
